# Dental hesitancy: a qualitative study of culturally and linguistically diverse mothers

**DOI:** 10.1186/s12889-022-14513-x

**Published:** 2022-11-28

**Authors:** Kanchan Marcus, Madhan Balasubramanian, Stephanie D. Short, Woosung Sohn

**Affiliations:** 1 Population Oral Health, Faculty of Medicine and Health, The University of Sydney School of Dentistry, Surry Hills, NSW Australia; 2 Health Care Management, College of Business, Government and Law, Flinders University, Adelaide, Australia; 3 Menzies Centre for Health Policy and Economics, School of Public Health, The University of Sydney, Camperdown, NSW Australia

**Keywords:** Oral health, Access, Healthcare disparities, Qualitative, CALD, Dental hesitancy

## Abstract

**Background:**

Oral healthcare is paramount and inextricably linked to well-being. Yet, the evidence indicates that culturally and linguistically diverse (CALD) migrant communities have unequal access to mainstream dental services due to several barriers. The purpose of this study was to investigate the oral healthcare experiences, attitudes and barriers to oral healthcare utilisation in CALD mothers.

**Methods:**

A qualitative study with semi-structured interviews was conducted within a social constructivism epistemology. CALD mothers who identified as non-English speaking, foreign country born, with a child under 12, were recruited though purposive snowball sampling. Questions probed oral healthcare experiences, barriers, enablers, and attitudes. Verbatim typed transcripts were thematically analysed using grounded methodology.

**Results:**

Thirty-three CALD mothers participated; twenty from India, five from Fiji, four from China, two from Nepal and one each from Israel and Macedonia. Languages included Cantonese, Fiji-Hindi, Gujrati, Hebrew, Hindi, Kannada, Mandarin, Maharashtrian, Macedonian, Nepalese, Punjabi, Sanskrit, Telegu and Urdu. **C**ost was the foremost barrier to oral healthcare services, followed by **C**onfidence in quality care for the provision of services and treatment. **C**onfusion in navigating a public and private healthcare system was highlighted and **C**ompeting priorities took precedence. **C**omplacency referred to ‘no need’ or lack of urgency in dental care. Subsequently, dental hesitancy (superordinate theme) described the patterning of data as comprising the five ‘C’ factors and was theorised as the *dental hesitancy phenomenon* to explain the occurrence of delay or avoidance in utilising dental care.

**Conclusion:**

Findings highlight the utility of the *dental hesitancy phenomenon* unearthed within this study. CALD mothers explained five ‘C’ dimensions: cost, confidence, confusion, competing priorities and complacency as barriers to accessing timely dental care. Multisectoral collaboration between healthcare systems, universal health coverage and primary sector support is required to address dental hesitancy in CALD mothers. Further, this study contributes to the field of behavioural and social sciences in oral health and augments the literature on dental avoidance.

**Supplementary Information:**

The online version contains supplementary material available at 10.1186/s12889-022-14513-x.

## Background

Oral healthcare inequities and inequalities are widely reported amongst populations [1]. The evidence emphasises that the mouth is linked to non-communicable risk factors and therefore, promoting prevention in dental care is prominent to stem population health inequalities [2]. The WHO resolution in oral health declared that Universal Health Coverage (UHC) contributes towards achieving Sustainable Development Goals for ‘good health and well-being’ and ‘reducing inequalities’ [3]. However only a handful of countries currently incorporate UHC in oral healthcare, with Australia lagging behind comparable European countries. A systematic review conducted in Europe, affirmed that migrant populations utilised emergency services for oral healthcare more than host country counterparts [4]. Migrants, or what the authors classify as culturally and linguistically diverse (CALD) populations experience multiple barriers in language, literacy, sociocultural norms, policy among other factors [5, 6]. This then intensifies inequalities in oral healthcare experienced by CALD communities.

Health sociologist Sarah Nettleton [7] identified motherhood discourses as the creator and controller of the domestic environment in a sociological study. This study conceptualised four discourses which described mothers as either ‘natural’ ‘ignorant’ ‘responsible’ or ‘caring’ dental agents for government. To exemplify, the home was considered a useful target population for government campaigns [7]. In essence, the discourses highlighted diverse beliefs and social experiences which influence a mother’s ability to act as dental agents for their child. Hence, similar constructs occur for CALD mothers, who migrate from specific historical, social, economic and political circumstances which multiplies power, agency or on the contrary, vulnerabilities [8]. The Australian Bureau of Statistics reported over six million mothers and 7.3 million families reside in Australia [9]. Almost 30 % of the population was overseas born and nearly half the Australian population were classified as CALD in 2021 [10]. According to the Census, over 300 languages are spoken in Australia [11], reflecting the importance in understanding population-specific oral healthcare needs for CALD mothers. In the state of NSW, 60 % of the population utilised dental services annually [12], but the utilisation of dental care is not culturally shared amongst all population groups. In this manner, the concept of prevention in oral healthcare, that is, directed towards maintaining lifelong oral health and teeth is not mutually shared. It is unclear why and/or which factors hinder CALD populations.

Quite a considerable literature investigates ‘dental avoidance’ to explain the psychosocial fear that inhibits oral healthcare utilisation [13]. For instance, Armfield and Ketting revealed that adults presenting with high level anxiety encountered additional barriers in affordability and communication to dental healthcare in Australia [14]. However, few studies have explored oral healthcare experiences in CALD migrant populations beyond psychosocial factors. In a qualitative study undertaken with CALD mothers in Melbourne, Australia, accessibility, fees, waiting lists and problematic interpreter experiences were affirmed [15]. Yet, fundamentally, CALD mothers experience various, multiple influences in the country in which they live. System and policy barriers, including long public dental waiting lists as well as negative provider experiences and an illness approach to dental care are some known barriers for CALD mothers [16]. Moreover, it is unclear whether intra-variability exists in oral healthcare access across CALD communities, given the limited number of qualitative studies conducted within an Australian context. For instance, in Western Australia, interviews with African migrant carers were conducted in an aged care setting [17]. This study highlighted limited prevention knowledge and dental visitations for pain [17], however, only ten female carers were interviewed. Moreover, much remains unknown about CALD mothers and their oral healthcare experiences. Therefore, the purpose of this study is to explore the experiences and attitudes to oral healthcare utilisation in CALD mothers residing in Australia. This study aim contributes towards the field of behavioural and social sciences in oral health and is aligned with the WHO resolution in oral health policy, with the broader objective to promote population equity.

## Method

The social constructivism paradigm (Vygotsky) informed the epistemological orientations of this study [18, 19]. We draw upon Creswell and Plano Clark’s constructivism definition as understanding knowledge through multiple participant meanings [20], and experiences through social and cultural interactions [19]. That is, CALD mothers are influenced by their environmental and sociocultural fabric when accessing and utilising dental services, and in this context, co-create meaning and knowledge. The broad research team comprised epidemiologists in oral health and behavioural and social scientists. Specifically, the first author is a CALD mother and experienced qualitative researcher, for which purposes we consider, the ‘insider-outsider’ positioning. Instead, the author self-situates along the ‘space between’ rather than a dichotomy [21]. Belonging within a CALD group potentially reinforces shared meanings or habits with participants, which could be measured through shared ‘language’. This is also advantageous in understanding the historical and cultural facets from participant voices [21]. Despite the positioning, self-reflexivity and researcher bias was adhered for all interviews. Co-authors of this study were positioned as ‘neutral’ considering interviews were conducted by the first author.

### Qualitative sample and CALD definition

An epidemiological review by Pham and colleagues [22] suggested a minimum of two defining variables for CALD related research. Individuals born in non-English speaking countries and those whose first language is not English. Supported by this classification [23], foreign born mothers from a non-English speaking country, who conversed in a non-English language were recruited. While interpreters were offered, CALD mothers who consented to participate displayed a level of English proficiency and all declined the need for an interpreter. Participants also had at least one child under the age of twelve.

### Sampling and data collection

A purposive snowball sampling method was undertaken. In 2016, the state of NSW comprised 30% of Australia’s total migrant population [24]. Demographically, CALD mothers were recruited from the Greater Western region of Sydney. This is home to the largest multicultural population with nearly 40% of households who converse in a non-English language [25]. Recruitment occurred through CALD community groups and Facebook CALD communities. Emails and private messages were sent to administrators of Facebook CALD community groups. Administrators had the discretion to disseminate study information to their community networks. CALD mothers were invited to participate in a one-one interview about their oral healthcare experiences. Semi-structured interviews were conducted in English either by phone, Zoom or in person which lasted between 25 and 60 minutes in duration. Interviews were conducted between March 2021 – July 2021. Participant information, consent and questions were all addressed prior to the conduct of interviews and voluntary participation was emphasised. Semi structured questions explored the oral healthcare experiences of CALD mothers in the origin country and in the current Australian system. Questions probed dental utilisation attitudes and perceptions, historical experiences of dental care, accessibility to services, provider experiences and barriers and facilitators to oral healthcare in Australia. A $20 gift card was presented to all participants, including CALD mothers who withdrew from the study; due to children/time (*n* = 2) and those that did not meet eligibility criteria (*n* = 2). An Olympus digital recorder was utilised, and transcripts were typed verbatim in Word. Ethical approval was granted by the University of Sydney Human Research Ethics Committee (2021/101).

Researcher bias was minimised through iterative coding and ongoing reflections of the researcher [26]. This was achieved with mind mapping, self-reflections, memos, journaling and the interrogation of unintended bias for the duration of this study. Further study rigour was aligned with fit, whereby the data applied to participant responses and applicability, that is, findings offer new insights regarding dental utilisation [27]. Additionally, the developed themes were created from the properties and dimensions within the data and historical contextualisation for CALD mothers were also collated to understand previous dental care experiences [27]. Study reliability and triangulation was conducted with the research team through repeated discussions of the data, coding and analysis [28]. Quotations were selected to include as many participant voices as possible.

### Data analysis

Data analysis was performed using grounded methodology within the school of Strauss and Corbin [27]. Grounded theory in this study emphasises a research method and generation of theory, to explain the experiences of dental care in CALD mothers. Inductively, line by line open coding with reiterative reading, memoranda and repeated examination of transcripts was performed. Coded data (which included demographics, experiences etc.) was sorted into categories using Excel (known as axial coding). Sorted categories were then grouped into sub-themes of shared similarities, patterns or experiences. This process was cyclical, ongoing and repeated over a period of ten months. Theoretical data saturation was achieved when no further insights or new data emerged with CALD mothers interviewed [27]. Five codes were removed from further analysis, which occurred due to lack of group consensus. For instance, the code ‘fear’ was dropped, as reported by only one participant. Subsequently, sub-themes were identified from coded data which resulted in two overarching themes; 1) dental hesitancy and 2) experiences with health workforce personnel. For the purposes of this paper, dental hesitancy is extrapolated.

Following the above analysis, a theory was generated to describe the factors to oral healthcare utilisation in CALD mothers, which was grounded in the data. To situate this theory within the evidence base, a modified version of the vaccine hesitancy model was utilised, which was first devised by the WHO SAGE working group [29, 30]. This model highlights barriers to vaccine uptake which were surprisingly comparable to our data for dental care. The 3C vaccine hesitancy model incorporates confidence, defined as trust and safety, complacency such as self-efficacy and convenience, which encompasses affordability and literacy levels [29]. We diverge from ‘convenience’ in the vaccine model to incorporate ‘C’ factors based on our findings from within our data. Therefore, ‘5C’ sub-themes were included, which deviates to the ‘3C’ vaccine model (Additional file 1: Appendix 1). Notably, we distinguish our definition of ‘hesitancy’ from the SAGE working Group who ascertain ‘the delay or refusal, despite availability’. Instead, we define hesitancy in terms of the dictionary defined ‘reluctance or delay’. Notably, this model has not yet been applied within the oral healthcare literature, which has primarily focussed on behavioural aspects, or the five ‘A’ framework of access [31].

## Results

The resultant sample included 33 CALD mothers, who conversed in various languages and were born in either; China, Fiji, India, Israel, Macedonia and Nepal participated (see Table 1). Mothers had children aged 12 years or under. Sub-themes are shown below, as reported in order of frequency.

**Table 1:** Sample demographics of CALD mothers, *n* = 33

Pseudonym	Country of birth	Languages spoken other than English	Year migrated to Australia
Jia Li	China	Cantonese, Mandarin	2012
Li na	China	Mandarin	2017
Mei Zhen	China	Cantonese, Mandarin	2006
Xiang	China	Cantonese, Mandarin	2006
Aishwarya	Fiji	Fiji-Hindi	1995
Manushi	Fiji (Lautoka)	Fiji-Hindi	2013
Priyanka	Fiji (Lautoka)	Hindi	1990
Shreya	Fiji	Fiji-Hindi	1987
Sushmita	Fiji	Fiji-Hindi	2009
Aditi	India	Hindi, Sanskrit	2016
Amruta	India	Marathi	2009
Neha	India	Hindi	2003
Shraddha	India	Hindi	2010
Sulaksha	India	Hindi, Telegu	2009
Bipasha	India (Bangalore)	Hindi, Kannada, Telegu	2008
Isha	India (Bangalore)	Kannada	2009
Kalpana	India (Bangalore)	Hindi, Kannada	2014
Keerthy	India (Chennai)	Tamil	2020
Chhaya	India (Delhi)	Hindi	2019
Vaishali	India (Delhi)	Hindi	2009
Disha	India (Gujrat)	Gujrati	2010
Janki	India (Gujrat)	Gujrati	2009
Sujathaa	India (Hyderabad)	Hindi, Telegu, Urdu	2017
Shweta	India (Mumbai)	Hindi, Marathi	2008
Jasleen	India (Punjab)	Punjabi	2017
Amneet	India (Punjab)	Punjabi	2016
Gurpreet	India (Punjab)	Punjabi	2014
Sachpreet	India (Punjab)	Punjabi	2008
Surjit	India (Punjab)	Punjabi	2015
Aliza	Israel	Hebrew	2015
Vera	Macedonia	Macedonian	1996
Anushka	Nepal	Nepalese	2005
Rita	Nepal	Nepalese	2014

### Cost

Affordability was the foremost barrier to oral healthcare utilisation in our study. In consensus, dental care in Australia is *extremely costly (Amruta, India)* for CALD families. A Medicare system was suggested by mothers, to help cover the cost of general cleaning. This was particularly highlighted when arriving from a foreign country in which the currency conversion rate is much lower to the Australian dollar *… It is too much … especially when you migrate initially-in the initial stages, every dollar looks very big (Amruta, India).*

*Uh, (dental access) problems, because of it being expensive! Then I said, “Okay”. This Australia, this Nepal, the condition seems similar … (Rita, Nepal).*

Knowledge, attitudes and belief in regular dental visits to prevent severe oral health problems from occurring was generally shared by 60% of CALD mothers. This idea of preventive oral healthcare seemed to be well understood. Although CALD mothers displayed an understanding and knowledge about oral healthcare prevention, the delay in access and utilisation occurred, as predominately related to the costs involved.

*People from our background, from India, we do delay the treatment. We don’t try to seek that care which we should just because of the cost involved. We’re trying to heal or treat ourselves on our own. We are just taking painkillers but not taking good care or health care and not accessing, not seeking the dental care treatment or the cleaning services or the dental check-up that should be done....(Amneet, India).*

One solution to this issue of cost was dental travel to the home country. However, this largely related to multiple factors of family support, lower costs and/or known, trusted providers in the home country.

*You know, you have to spend a lot, and then you’ll be like, “oh I’ll leave it this year, we can do it next year”. Something like that, I have like, five fillings to be done, you know … And then you’ll be like, oh, if I can avoid, you know, then you’ll avoid it. That’s one thing which I think is really expensive. You have to go back in India. It’s very cheaper over there...(Disha, India).*

### Confidence in quality care

Confidence was reported by participants as the desire for trustworthy, quality care providers, reiterated in terms of an ethical or ‘good dentist’. Some CALD mothers reported negative dental experiences with an inadequate provision of care in Australia (and/or in the home country) that occurred to the CALD mother and/or their families. The below excerpt is from a dental care experience that occurred in Australia.

*But yeah, they (relative) were not cared for properly. And so, I just don’t know if there could be a regulatory body in that space as well to make sure the treatment which the dentist provides is up to the mark … She was unhappy with the dentist. And she said that yeah, it’s not worth it, you know, going and visiting a dentist. I keep up with the regular treatment but then there are things which are not right …*. *then you know, they stop having trust. So, that becomes a trust issue then, then you don’t want to go and see a dentist and then same thing you pass on to your kids … (Vishali, India).*

Dental provider trust was further underscored whereby treatment decisions were enforced onto the patient. This however posed concerns about need and/or unnecessary work. For instance, a mother described unnecessary treatment, although she was unable to question the dentist at the time.

*… kind of enforced, and you kind of felt like, you said it was, sort of, pushed into doing it … And then, you see it’s- when you’re sitting on the chair, you feel, worried, [chuckles] mouth wide open...That is not the time where you can say a lot of things...No, we can leave it in-I’m happy with that or things like that. Uhm, and she (Dentist) … “Okay, so you’ve got this black thing here. So, let’s get it out and I’ll just replace it with something else.” I’m like, Uhm, okay....uhm, I’m happy with what I’ve got, I don’t mind it. I’d rather leave it in … (Amruta, India).*

Dental billing concerns were raised whereby dental practices have the discretion to decide the fee amount, which invariably differs by each surgery. Some practices charged consultation fees or provided unnecessary work. CALD mothers who encountered such experiences, expressed avoidance of the specific dental provider/clinic for future dental visits for their own family. A mother reported that she was forced for an x-ray at a new clinic, when she already retained a recent x-ray from a previous clinic and didn’t wish to expose herself to radiation superfluously. The lack of standardization in billing or policies at provider centres was further questioned by CALD mothers in terms of patient interests or business profits.

*I tried to ask why and why there is a gap...Somebody told me they took some deep clean, or they have some x-ray done...I’m not so sure whether they done* … *but I’ve still been forced to do that (x-rays*) or *why are you (dentist) just gonna charge me for the $180 for the first consult? (Xiang, China).*

Experiences of dissatisfaction with dental providers was also reaffirmed with a sense of frustration.

*I think the way, way we were charged. Because they both say they did cleaning. But I don’t know what sort of cleaning they did. So, because cleaning I didn’t, I didn’t ask you to do cleaning, why you’re doing it? They charged us a hundred dollars for it, which was not needed. So, I didn’t like that. (Janki, India).*

### Confusion navigating the public-private healthcare system

The Australian healthcare system was described as confusing, particularly regarding the dual public-private healthcare system and lack of information. Uncertainty about where to begin within the dental system was reported *… .though I’m working here, I don’t have much connectivity or knowledge about the local dentistry over here (Keerthy, India).* The healthcare system in the home country was described as ‘easier’ to understand compared to the ‘confusing’ and *very difficult to understand the difference between public and private (Sushmita, Fiji)* healthcare system in Australia. This perception was shared between majority (75% of participants), including mothers with tertiary qualifications and spoke with high levels of English proficiency. Co-designed suggestions were provided by mothers, for instance simple, graphical information (translated into other languages) that entails system information, and what is covered and where to access support*.* Navigating the healthcare system was more pronounced for new mothers to Australia, compared to mothers residing in Australia for over ten years.

*...I think it might be a bit confusing when you- like the Australian system is- is not- it’s not private but it’s not public. It’s something in between. In Israel, it’s very uh, public uhm, more public. So, it was a bit confusing in the beginning to understand what is included in the insurance, what is not. Uhm, whose doctor work- works with which insurance? First, when you emigrate, you prefer uhm- [clicks tongue] you prefer not to have any out-of-pocket expenses.” (Aliza, Israel).*

*Maybe there should be a service where they can let you know what you’re, just like a Medicare thing...When they can let you know what you are, you know, ah, ah, you know entitled to and how to access some of the services. And I know, I know that some hospitals do you have dental care but you need to be on a waiting list ...I think this is only if you’re getting umm Centrelink payment.... But it’s almost like umm, it’s almost like a hush-hush...Like, people don’t really know about it. (Vera, Macedonia).*

A key distinction to the Australian oral healthcare system and the origin country was the need to plan dental visits approximately six months in advance. In this example, a mother referenced a specific provider who was associated to a private insurance company, which meant that no gap payment would be incurred. This experience was starkly different to healthcare systems from origin countries, with next day availability of dental appointments or walk-in clinics. Oral healthcare information was also requested by CALD mothers who lacked social and cultural support and/or were new to Australia (under 6 years).

*In China if you feel some prob-problem, you can see the dentist uh right away more quickly, yeah, yeah, yeah. It’s different. Yeah, and here, I think normally immigrants don’t know you need to arrange this thing in advance yeah. Like, like you arrange a holiday, like few months before, yeah, it’s different. I think it’s the different culture. (Li na, China).*

### Competing priorities

Starting anew in a foreign country required various changes in the lifestyle, system and processes. The Australian lifestyle was constantly reaffirmed as ‘busy’ and ‘hard’ in terms of juggling all else, which resulted in the delay in access or utilisation of oral healthcare services for CALD mothers. Child-family responsibilities also took precedence whilst time challenges added to the complexity when working full time or negotiating care for children …. *And having young kids, going to the dentist by myself is really, a really hard thing um, because I’m pretty much primary carer of the kids, so, it’s, it’s like I would need to organise babysitting to go to the dentist. (Shreya, Fiji).*

Mothers reiterated *the focus is on the children. You can’t get dental and general well-being, and like you always pushed yourself to the side (Priyanka, Fiji).* A regular dental clean/check-up was also not a priority due to competing health concerns that were considered more significant than oral healthcare. *You know, there are many other more important issues to handle like day-to-day issues. My diabetes, you know, uh, my weight and stuff like that which I need to be in control. (Keerthy, India)*.

### Complacency

Complacency encapsulated the concept of ‘no need’, self-efficacy and lack of oral health priority. Oral healthcare avoidance due to sociocultural beliefs of ‘no need’ and the perceived risk from a lack of regular oral healthcare was considered low. Sociocultural practices in origin countries differed, for which dental care was likely to be obtained when pain was experienced by 36% of participants. Dentist access or utilisation was further avoided if pain subsided. Several access barriers overlapped which led to dental delay or avoidance, as described by CALD mother, Gurpreet. The below example underlines differing sociocultural factors in terms of health education for prevention as well as system related policies to enable reduced oral healthcare costs.

*… one is no need. I don’t have any pain and the-- otherwise like ah it’s costly as well. So, I don’t want to go for like just for normal check-up. (Gurpreet, India).*

Another aspect of complacency involved difficulties in behaviour change due to the multiple demands, time, norms or habits associated with living in Australia.

*...honestly until now, I have never been to a dentist for myself … They (people from community) don’t go to the dentist unless they are under severe pain. They postpone things. Okay, let’s go in tomorrow. It’s not ringing, paining or they’ll try to home medicine, then clove oil … They’ll have to wait and see whether it’s going up ... And then only at the later stage they will go to the doctor. Even if it comes to me, I will do the same though. It’s very hard for me to change myself. Um every time I say. Okay. I think I’ll do it from next week. I’ll do it from next week. Always from next week, but I,I,I don’t know how to do it along with how I’m living, you know. (Sulaksha, India).*

**Fig. 1:**
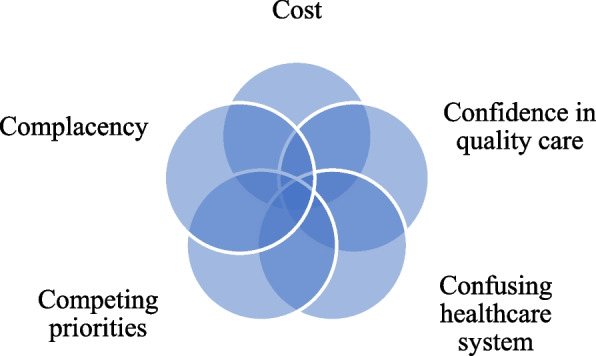
The dental hesitancy phenomenon

**Fig. 2:**
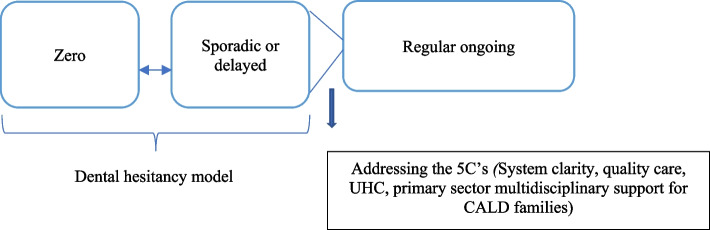
Dental utilisation from zero to regular oral healthcare utilisation in CALD mothers

Several individual, social and system factors overlapped and interconnected. For instance, cost and competing priorities overlapped when discussing life and child caring responsibilities. Confidence incorporated trustworthiness and provider competence while complacency involved the lack of priority given to oral healthcare. Hence, the interplay between macro systems, provider and individual/family level factors align with the previous evidence [16]. On a separate note, acculturated (that is, the duration of time exposed to host country norms and practices) CALD mothers who resided in Australia for over ten years, also expressed dental hesitancy due to cost, lack of time or need barriers. Our study suggests that CALD mothers from India were more likely to travel to the home country for family purposes and consequently receive dental care, which was not reiterated by other country born CALD mothers. Significantly, CALD mothers asserted hesitancy with the delay or avoidance in dental care services due to five 'C' determinants within the Australian context. Collectively addressing all dimensions of the five 'C’s would facilitate dental access and utilisation for CALD mothers (Fig. 1). Our social constructivist conceptual approach enables us to explain concepts, constructs and relationships to coherently explain the phenomenon of interest [32], in this case, dental utilisation in CALD mothers. The combination of five ‘C’ factors and the theme, dental hesitancy was together theorised as the *dental hesitancy phenomenon*. This phenomenon explains the barriers to underutilisation or dental delay for CALD migrant mothers as a result of five 'C' factors, as illustrated in Fig. 2. We utilised a continuum to define hesitancy as never having accessed dental care to sporadic, delayed dental care. Notably the *dental hesitancy phenomenon* was both inductively and deductively devised from within the study data.

## Discussion

This study contributes firsthand experiences in oral healthcare for CALD mothers residing within the Australian multicultural setting. Findings highlight the utility of the *dental hesitancy phenomenon* within the Australian context. The phenomenon is used to explain both the dental hesitancy theme and the five ‘C’ determinants as barriers to accessing oral healthcare: cost, confidence, confusion navigating the healthcare system, competing priorities and complacency. Multiple intersecting factors that contribute to micro and macro level disparities in oral healthcare were evident. For instance, information clarity is required about the public-private healthcare system, particularly for new CALD mothers to the country. Illustrated by a CALD mother was the distinct sociocultural practices from the origin country to Australian norms. Metaphorically, this was depicted as the need to schedule dental care several months in advance, likewise, to planning a holiday. Hence, multipronged approaches at the policy, provider, community and family level that address the *dental hesitancy phenomenon* is required, to promote oral healthcare utilisation for CALD mothers. Despite the in-depth exploration of findings, caution is required when interpreting results. Our sample comprised mothers with an adequate to high level of English proficiency, despite various methods to recruit diversity in languages and countries. Moreover strengthening policy, programs and community support, in alignment to the WHO UHC resolution in oral healthcare [3] to promote oral healthcare equity in CALD communities is needed within the Australian context.

The utility of this *dental hesitancy phenomenon*, from the perspective of CALD mothers, shifts beyond the psychosocial fear focus within the behavioural oral healthcare literature. A wider range of sociocultural factors hindered access to dental services within this Australian context and time (We note time, considering publicly funded dental care programs are limited at present, but this could change in the future). Notably, the complexity in accessing dental care is not merely individual level factors. Social factors interact and hinder dental accessibility. Thus, logistical challenges were reported although this did not relate to geographic proximity of dental providers in our findings. For example, CALD mothers who were employed during weekday hours were coupled with home responsibilities after hours. Therefore, dental hesitancy is not solely a behavioural or psychological issue, which contrasts with the evidence on dental anxiety [33, 34]. In this case, CALD mothers do not appear to have experienced traumatic childhood dental experiences, that is commonly linked to dental anxiety [34]. Distinctly, the five ‘A’ framework; approachability, acceptability, accommodation, affordability, and appropriateness, was not utilised considering all these domains were not reported in our study data. To further elucidate, participants interviewed were in urban geographic locations and the expansion of dental practitioners across greater metropolitan areas meant that ‘accommodation’ was irrelevant in our study findings.

The *dental hesitancy phenomenon* is aligned to widely cited evidence of social determinants, whereby individual living and working (and other) conditions consequently impacts health [2]. This contrasts to the literature on vaccination hesitancy [29] [35]. For instance, vaccination hesitancy is not necessarily impacted by education or socioeconomic status wherein low levels of vaccine uptake is indicated in highly educated groups [36]. For instance, Sabbah and colleagues [37] conducted an epidemiological study in oral health, using the National Health and Nutrition Examination Survey (NHANES) data. The study substantiates a social gradient in oral health which impacts health outcomes, as influenced by income and education [37]. Further, Lane and colleagues reported a global analysis which explored the most common reason for vaccine hesitancy. Findings underline the ‘risk-benefit’ parameter as the main hinderance to vaccine hesitancy [38]. This ‘risk-benefit’ can be viewed in congruence to ‘complacency - competing priorities’ from our study. Parallels are moreover evident in vaccine miscommunication and the lack of accessible trustworthy oral healthcare information, which remains an ongoing challenge to be addressed, particularly for CALD communities [23]. For instance, one cross sectional study identified disparities in trust pertaining to vaccine hesitancy amongst CALD groups [39]. Associated with trust, confusion is a reported barrier in both vaccine and dental hesitancy. In our study, CALD mothers narrated confusion in navigating the oral and general healthcare system, including subsidised dental care options. The OECD substantiates that Australia’s healthcare system is one of the most complicated systems in the world [40] and thus healthcare system simplicity and information clarity is required. Consequently, implications for the lack of public oral health information and/or messaging interposes with the dimension of complacency and the inappropriate perpetuation that oral health is not a public health priority. Targeted communication in collaboration with CALD communities, including a ‘one-stop’ online website (with simplified information) and primary healthcare professionals was suggested by participants as key enablers to overcome dental hesitancy.

### Limitations

Findings from this study lack transferability to other contexts or population groups, due to the specific sample of CALD mothers interviewed. Outlying data unfit for inclusion within this dental hesitancy theme included CALD mothers who were unable to travel to the home country because of COVID related travel restrictions. This comprised 18% of the sample, who primarily travel for family purposes and then subsequently receive dental treatment. Reasons for overseas dental treatment generally intertwined with cost, confidence in quality care and competing priorities and thus was not included as a sub-theme. Considerations for study limitations included distinct socioeconomic positions of CALD mothers. For instance, mothers who utilised private health insurance to help reduce dental costs, in contrast to mothers without the ability to pay for dental services and treatment. This exacerbates oral healthcare inequalities whereby governing actors and regulating bodies have the power to influence and shape population health equity. Participant recruitment inequities occurred within this snowball approach whereby online membership to CALD specific community groups automatically excluded participants without access to this form of social and cultural capital. Language barriers were not reported in this sample, which is likely to differ to the needs of humanitarian and refugee CALD migrants. Perceived or experiential racism was not probed as participants in this study reported ‘respectful’ experiences. Again, this could be attributed to participants displaying English proficiency, tertiary qualifications and/or employment/income levels. More specifically, we did not collect qualification/income data as per ethical approvals, however just under half of the group voluntarily reported this information. The evidence indicates racial inequalities are experienced by differing population groups and thus further research with refugees and those with low levels of English proficiency is warranted. Implications from this study help inform oral healthcare services that are relevant for CALD communities. Further research is needed to assess the comprehensiveness of findings. This could include constructs in quantitative research methods that address questions pertaining to the 5C’s.

## Conclusion

Understanding the reasons for low uptake of oral healthcare utilisation in CALD communities, with first generation migrants whose first language is not English, is essential to promote population health equity. Findings from this study reveal the utility of the *dental hesitancy phenomenon* as the delay or avoidance in access or utilisation of oral healthcare in CALD mothers. This phenomenon incorporates the combination of the dental hesitancy theme and attributing five ‘C’ factors: cost, confidence in quality care, confusion navigating the healthcare system, competing priorities and complacency. Multisectoral collaboration to address all ‘C’ factors is required. Explicitly, integration of the primary and oral healthcare systems, including policy development for improved and targeted programs and services, health education support and the provision of accessible and simplified information. Findings further support universal health coverage in oral health within the Australian context, to promote more equitable access to oral healthcare within CALD communities.

## Supplementary Information

**Additional file 1.**

## Data Availability

The datasets used and/or analysed during the current study are available from the corresponding author on reasonable request however, all the data generated or analysed during this study are included in this published article [and supplementary Appendix file].

## Abbreviations

CALD: Culturally and linguistically diverse
NSW: New South Wales
UHC: Universal Health Coverage
WHO: World Health Organisation

## References

[1] Watt RG, Daly B, Allison P, Macpherson LMD, Venturelli R, Listl S (2020). The Lancet Oral Health Series: Implications for Oral and Dental Research. J Dental Res.

[2] Peres MA, Macpherson LMD, Weyant RJ, Daly B, Venturelli R, Mathur MR (2019). Oral diseases: a global public health challenge. Lancet.

[3] WHO. Oral Health: Achieving better oral health as part of the universal health coverage and noncommunicable disease agendas towards 2030. 2020 23 December. Contract No.: EB148/8.

[4] Pabbla A, Duijster D, Grasveld A, Sekundo C, Agyemang C, van der Heijden G (2020). Oral Health Status, Oral Health Behaviours and Oral Health Care Utilisation Among Migrants Residing in Europe: A Systematic Review. J Immigrant Minority Health.

[5] Amin M, Perez A (2012). Is the wait-for-patient-to-come approach suitable for African newcomers to Alberta, Canada?. Commun Dentistry Oral Epidemiol.

[6] Mariño R, Wright C, Schofield M, Minichiello V, Calache H (2005). Factors associated with self-reported use of dental health services among older Greek and Italian immigrants. Special Care Dentistry.

[7] Nettleton S (1991). Wisdom, diligence and teeth: discursive practices and the creation of mothers. Sociol Health Illness.

[8] Muirhead E, Milner A, Freeman R, Doughty J, Macdonald M (2020). What is intersectionality and why is it important in oral health research?. Commun Dentistry Oral Epidemiol.

[9] ABS. Media Release: Happy Mother's Day from the ABS! Canberra 2018 [updated 10 May. Available from: https://www.abs.gov.au/ausstats/abs@.nsf/mediareleasesbyReleaseDate/168BFDA0C45F98A8CA258288001A58C5?OpenDocument.

[10] ABS. Cultural diversity: Census Information on country of birth, year of arrival, ancestry, language and religion Canberra 2022 [Available from: https://www.abs.gov.au/statistics/people/people-and-communities/cultural-diversity-census/latest-release.

[11] ABS (2017). Cultural diversity in Australia.

[12] Marcus K, Balasubramanian M, Short S, Sohn W (2022). Cultural and linguistic disparities in dental utilisation in New South Wales, Australia. Community Dent Health J.

[13] Armfield JM, Stewart JF, Spencer AJ (2007). The vicious cycle of dental fear: exploring the interplay between oral health, service utilization and dental fear. BMC Oral Health.

[14] Armfield JM, Ketting M (2015). Predictors of Dental Avoidance Among Australian Adults With Different Levels of Dental Anxiety. Health Psychol.

[15] Riggs E, Gussy M, Gibbs L, van Gemert C, Waters E, Kilpatrick N (2014). Hard to reach communities or hard to access services? Migrant mothers' experiences of dental services. Aust Dent J.

[16] Marcus K, Balasubramanian M, Short S, Sohn W. Barriers and facilitators to dental care among culturally and linguistically diverse carers: a mixed methods systematic review. Community Dent Oral Epidemiol. 2022:1–24. 10.1111/cdoe.12745. 10.1111/cdoe.12745 35342972

[17] Adebayo B, Durey A, Slack-Smith LM (2017). Culturally and linguistically diverse (CALD) carers' perceptions of oral care in residential aged care settings in Perth, Western Australia. Gerodontology.

[18] Crotty M. The foundations of social research: meaning and perspective in the research process. London: Routledge; 2020.

[19] Detel W. Social Constructivism. USA: Elsevier Ltd; 2001. p. 14264–7.

[20] Creswell JW, Plano Clark VL (2011). Designing and conducting mixed methods research.

[21] Katie K (2012). Insider, outsider, or somewhere in between: The impact of researchers’ identities on the community-based research process Journal of Rural. Soc Sci.

[22] Pham TTL, Berecki-Gisolf J, Clapperton A, O'Brien KS, Liu S, Gibson K (2021). Definitions of Culturally and Linguistically Diverse (CALD) : A Literature Review of Epidemiological Research in Australia. Int J Environ Res Public Health.

[23] Marcus K, Balasubramanian M, Short S, Sohn W (2021). Culturally and linguistically diverse (CALD): terminology and standards in reducing healthcare inequalities. Aust N Z J Public Health.

[24] ABS. Migration, Australia, 2018–19 Canberra: Government of Australia; 2021 [Available from: https://www.abs.gov.au/ausstats/abs@.nsf/Latestproducts/3412.0Main%20Features32018-19?opendocument&tabname=Summary&prodno=3412.0&issue=2018-19&num=&view=.

[25] ABS. 2016 Census QuickStats - Greater Western Sydney Canberra: Australian Bureau of Statistics; 2017 [Available from: https://quickstats.censusdata.abs.gov.au/census_services/getproduct/census/2016/quickstat/1GSYD?opendocument.

[26] Daly J, Kellehear A, Gliksman M (1997). The public health researcher.

[27] Corbin JM, Strauss A (2008). Basics of Qualitative Research: Techniques and Procedures for Developing Grounded Theory.

[28] Collingridge DS, Gantt EE (2019). The Quality of Qualitative Research. Am J Med Qual.

[29] MacDonald NE (2015). Vaccine hesitancy: Definition, scope and determinants. Vaccine.

[30] WHO. Report of the SAGE Working Group on Vaccine Hesitancy Turkey; 2014

[31] Penchansky R, Thomas JW (1981). The Concept of Access: Definition and Relationship to Consumer Satisfaction. Med Care..

[32] Lune H, Berg B (2017). Qualitative Research Methods for the Social Sciences.

[33] White AM, Giblin L, Boyd LD (2017). The Prevalence of Dental Anxiety in Dental Practice Settings. J Dent Hyg.

[34] Eli I, Uziel N, Blumensohn R, Baht R (2004). Modulation of dental anxiety - the role of past experiences, psychopathologic traits and individual attachment patterns. Br Dent J.

[35] Bedford H, Attwell K, Danchin M, Marshall H, Corben P, Leask J (2018). Vaccine hesitancy, refusal and access barriers: The need for clarity in terminology. Vaccine.

[36] Larson HJ, Jarrett C, Eckersberger E, Smith DMD, Paterson P (2014). Understanding vaccine hesitancy around vaccines and vaccination from a global perspective: A systematic review of published literature, 2007–2012. Vaccine.

[37] Sabbah W, Tsakos G, Chandola T, Sheiham A, Watt RG (2007). Social Gradients in Oral and General Health. J Dent Res.

[38] Lane S, MacDonald NE, Marti M, Dumolard L (2018). Vaccine hesitancy around the globe: Analysis of three years of WHO/UNICEF Joint Reporting Form data-2015–2017. Vaccine.

[39] Bagasra A, Doan S, Allen C (2021). Racial differences in institutional trust and COVID-19 vaccine hesitancy and refusal. BMC Public Health.

[40] OECD. Caring for quality in health: lessons learnt from 15 reviews of health care quality. Organisation for Economic Co-operation and Development; 2017.

