# Supplementary material for: Dental hesitancy: a qualitative study of culturally and linguistically diverse mothers
Source: BMC Public Health. 2022 Nov 28;22:2199. doi: 10.1186/s12889-022-14513-x (PMC9703727; doi:10.1186/s12889-022-14513-x)
Supplement: Supplementary file 1 — Additional file 1. [file 12889_2022_14513_MOESM1_ESM.docx]

**Appendix 1**

*Table 1 The overarching theme with sub-themes and codes as reported by CALD mothers*

| **Overarching Theme** | **Sub-Themes** | **Codes** |
| --- | --- | --- |
| *Dental hesitancy* | Cost | Expensive consultation and treatment (especially for new migrants to Australia) |
|  |  | Medicare universal health system for dental considered ideal, unknown public rebate-dental programs |
|  |  | No private insurance coverage to help with treatment cost / travel to home country for dental services |
|  |  | Information requested about subsidised dental schemes to help with cost: Child Benefit Dental Scheme |
|  |  |  |
|  | Confidence in quality care | Trust in dental health provider / Treatment didn’t resolve issue / Dissatisfaction with provider |
|  |  | Questionable unnecessary treatment / return visits not logical for CALD mother |
|  |  | Desire for ‘good’ dentist, as probed to mean specialised / ethical dentists / quality treatment provider |
|  |  | Dental billing concerns / provider clinics differ in charges & policies / lack of cost standardisation |
|  |  |  |
|  | Confusing healthcare system | Public-Private system confusion / where to begin / which is ‘best’ option |
|  |  | Distinct cultural norms to origin country - need to book dental care months in advance in Australia |
|  |  | New CALD migrants navigating complex system different to home country (“Need for referrals?”) |
|  |  |  |
|  | Competing priorities | Child-care / family responsibilities (includes grandparents) |
|  |  | Busy life here in Australia / constant juggling |
|  |  | Other issues are a priority / diabetes-weight examples |
|  |  | Working weekdays, dentist closes 5pm |
|  |  |  |
|  | Complacency | Sociocultural norms of ‘no need’ |
|  |  | Lack of childcare/family support to allow mother to seek dental care |
|  |  | Self-efficacy / mothers push own needs aside (majority of CALD participants had knowledge of preventive oral healthcare) |
|  |  | Pain disappears, so dental care is not considered urgent |
|  |  | Origin/home country lack of oral healthcare emphasis during school years – reinforces lack of urgency |
